# Concentrated Bioshell Calcium Oxide (BiSCaO) Water Kills Pathogenic Microbes: Characterization and Activity

**DOI:** 10.3390/molecules25133001

**Published:** 2020-06-30

**Authors:** Shingo Nakamura, Masayuki Ishihara, Yoko Sato, Tomohiro Takayama, Sumiyo Hiruma, Naoko Ando, Koichi Fukuda, Kaoru Murakami, Hidetaka Yokoe

**Affiliations:** 1 Division of Biomedical Engineering, National Defense Medical College Research Institute, 3-2 Namiki, Tokorozawa, Saitama 359-8513, Japan; ishihara@ndmc.ac.jp (M.I.); iroihsh@gmail.com (S.H.); naoandokoro@gmail.com (N.A.); khf05707@nifty.com (K.F.); 2 Division of Statistical Analysis, Research Support Center, Shizuoka General Hospital, 4-27-1 Kita-ando, Aoi-ku, Shizuoka 420-8527, Japan; sato.yoko.shiz@gmail.com; 3 Department of Oral and Maxillofacial Surgery, National Defense Medical College Hospital, 3-2 Namiki, Tokorozawa, Saitama 359-8513, Japan; taka01@ndmc.ac.jp (T.T.); murakami@ndmc.ac.jp (K.M.); yokoe@ndmc.ac.jp (H.Y.)

**Keywords:** calcium oxides, deodorization, disinfection, heated scallop-shell powder, microbicidal activity

## Abstract

Bioshell calcium oxide (BiSCaO) exhibits deodorizing properties and broad microbicidal activity. In this study, we examined possible utility of BiSCaO Water for that purpose. BiSCaO Water was prepared by adding 10 wt% BiSCaO to clean water and gently collecting the supernatant in a bottle. The same volume of clean water was gently poured onto the BiSCaO precipitate and the supernatant was gently collected in a bottle; this process was repeated fifty times. The produced BiSCaO Water contained nanoparticles (about 400–800 nm) composed of smaller nanoparticles (100–200 nm), and was colorless and transparent, with a pH > 12.7. In vitro assays demonstrated that BiSCaO Water eliminated more than 99.9% of influenza A (H1N1) and *Feline calicivirus, Escherichia coli* such as NBRC 3972 and O-157:H7, *Pseudomonas aeruginosa*, *Salmonella,* and *Staphylococcus aureus* within 15 min. We compared BiSCaO Water with the other microbicidal reagents such as ethanol, BiSCaO, BiSCa(OH)_2_ suspensions, povidone iodine, NaClO, BiSCaO dispersion and colloidal dispersion with respect to deodorization activity and microbicidal efficacy. The results showed that BiSCaO Water was a potent reagent with excellent deodorization and disinfection activities against pathogenic bacteria and viruses (including both enveloped and nonenveloped viruses).

## 1. Introduction

Calcium oxide (CaO) and calcium hydroxide (Ca(OH)_2_) produced from limestone are readily available and important inorganic compounds used in various industries as adsorbents, toxic-waste remediation agents, and alkalization agents. However, both reagents contain harmful impurities, and CaO is especially dangerous, because it easily generates high heat in response to hydration [1,2]. Although scallop shells have been used as food additives and are a readily available source of CaO and Ca(OH)_2_, most are discarded as industrial waste. These wasted shells accumulate on the shores of harvesting districts in Japan, causing serious problems such as offensive odors and soil pollution due to release of harmful materials from the shells [3]. The harmful materials in scallop shells can be removed by heating at a high temperature, and by grinding and sieving. Most commercially available heated shell powder products used as food additives are composed of Ca(OH)_2_, called bioshell calcium hydroxide (BiSCa(OH)_2_).

The main component of scallop shells is calcium carbonate (CaCO_3_), which is converted to CaO when heated above 800 °C. Heated scallop shell powder is composed mainly of CaO and is called “bioshell calcium oxide (BiSCaO)”, a material exhibiting broad antimicrobial activity against various pathogenic bacteria [3,4], avian influenza virus [5], heat-resistant bacterial spores [6], fungi [7], and biofilms [8,9,10]. In addition, BiSCaO is used as an additive to prolong the shelf life of food products [3,6]. CaO is easily converted to Ca(OH)_2_ by hydration with water. CaO hydration generates a base, which is likely one of the mechanisms allowing BiSCaO to exhibit disinfection activity. For example, the disinfection activity of the CaO towards both total viable bacterial cells (TC) and coliform bacteria (CF) is higher than that of Ca(OH)_2_ or sodium hydroxide (NaOH) solutions at the same pH [10,11]. BiSCaO suspension (0.2 wt%) has been used to disinfect contaminated wood and pig skin pieces to remove both TC and CF, and is more effective than the equivalent concentration of hypochlorous acid (HClO) (pH 6.5) and sodium hypochlorite (NaClO) (pH 9.5). BiSCaO suspension has higher disinfection activity than HClO and NaClO in terms of both TC and CF [11,12]. Ten-times-higher concentrations of povidone iodine and chlorhexidine gluconate are required for disinfection compared to BiSCaO [11,12]. Thus, BiSCaO has high potential in the medical care and food industries.

Several techniques have been developed for cleaning chronic infected wounds, leg ulcers, and pressure ulcers using water or saline at pH values of between 5.5 and 6.5 and at temperatures of between 35 °C and 45 °C [13,14,15]. Weakly acidic (pH 5.5–6.5) HClO solution generated by mixing NaClO and weakly acidic water or saline has excellent in vitro bactericidal properties [16,17]. Daily wound cleaning with HClO solution (pH 6.5) for 12 days decreased the *Pseudomonas aeruginosa* (*P. aeruginosa*) bioburden of infected wounds in *db/db* diabetic mice but wound healing was delayed [18]. Limiting disinfection treatment with both HClO and chitin-nanofiber sheet-immobilized silver nanoparticles (CNFS/Ag NPs) to 3 days [19,20,21] may suppress these negative effects on wound repair [22]. We previously showed that treating *P. aeruginosa*-infected wounds on hairless rats with BiSCaO suspension (0.2 wt%, pH 12.3) once daily for 3 days and covering the wound with CNFS significantly decreased the *P. aeruginosa* bioburden and enhanced healing [23].

BiSCaO itself is poorly water-soluble under strongly alkaline conditions (pH > 12.3). Consequently, water suspensions containing a high concentration of BiSCaO tends to plug spray nozzles due to precipitation [12,24]. We previously reported that this precipitation can be prevented by adding phosphate compounds such as sodium hydrogen phosphate (Na_2_HPO_4_) [25] or sodium polyphosphate (Na-polyPO_4_) [26] into the BiSCaO-containing water. These resultant BiSCaO dispersions and colloidal dispersions exhibited higher deodorization and microbicidal activities than BiSCaO suspension.

In this study, we explored the possibility to test whether water containing highly concentrated BiSCaO can be made using commercially available bioshell calcium oxide (BiSCaO; Plus Lab Co. Ltd., Kanagawa, Japan; in which over 99.6% CaO is included), since a highly concentrated BiSCaO is thought be more potent against various infectious viruses and pathogenic bacteria. We added 10 wt% BiSCaO to pure water in a bottle and then collected the supernatant. The same volume of pure water was gently poured onto the BiSCaO precipitate accumulated in the bottle and the supernatant was carefully collected. This process was repeated fifty times. The resultant BiSCaO suspension contained smaller nanoparticles (100–200 nm in diameter), and was colorless and transparent with pH > 12.7. We call this “BiSCaO Water”. This study is aimed to demonstrate that BiSCaO Water is effective for detoxicating various types of viruses and bacteria compared with conventional BiSCaO solutions, as we described previously. The BiSCaO Water may have a potential as antiseptic/disinfectant for various infections caused by serious pathogens [27].

## 2. Results

### 2.1. Characterization of BiSCaO Water

**Figure 1:**
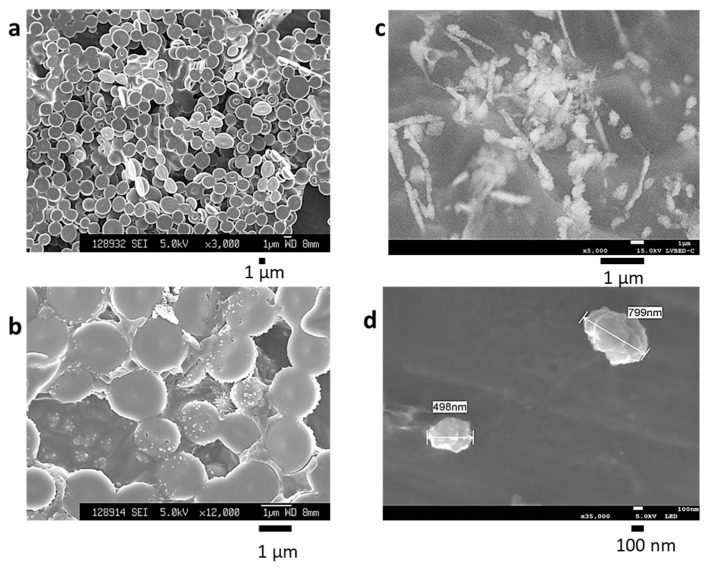
Scanning electron microscopy (SEM) images of drying BiSCaO Water and cryo-SEM images of BiSCaO Water. The particle surface structure of drying BiSCaO Water at 3000-fold magnification (**a**) and at 12,000-fold magnification (**b**) was observed with SEM images taken with a field-resolved scanning electron microscope. Cryo-SEM observations were performed on BiSCaO Water at 5000-fold magnification (**c**) and 35,000-fold magnification (**d**). Arrows indicate nanoparticles comprising assemblies of smaller nanoparticles.

Spraying BiSCaO Water, which is colorless and transparent with a pH > 12.7, on a smooth metal or plastic surface, followed by drying, provides a white powder coating. Scanning electron microscope (SEM) images of dried powders of BiSCaO Water are shown in Figure 1a,b, and cryo-SEM [22,28] images of BiSCaO Water are shown in Figure 1c,d. After drying BiSCaO Water, the BiSCaO particles become larger (1–2 μm) compared to those in BiSCaO Water. The microparticles were connected with each other. Nanoscale CaO particles (100–200 nm) in BiSCaO Water aggregate to generate larger BiSCaO particles (400–800 nm). Furthermore, when the dried powder was suspended into clean water, the powder was insoluble and pH in the supernatant was below 10.5, suggesting the microparticles were CaCO_3_ produced by interaction of Ca^2+^ and CO_2_.

### 2.2. Assay for the Disinfection Activity of BiSCaO Water against Pathogenic Bacterias and Viruses In Vitro

Bactericidal activities of BiSCaO against *Escherichia coli* (*E. coli*) NBRC 3972, *E. coli* O-157:H7, *P. aeruginosa*, *Salmonella,* and *Staphylococcus aureus* (*S. aureus*) were evaluated by the Japan Food Research Laboratory (JFRL; Tokyo, Japan) (Table 1). The levels (colony forming units; CFU) of *E. coli*, *P. aeruginosa,* and *Salmonella* were reduced to below the detection limit within 5 min, 99% of *E. coli* O-157:H7 was eliminated after 5 min, and 97% of *S. aureus* (a Gram positive bacterium) was eliminated after 5 min and reduced to below the detection limit after 15 min.

The virucidal activities of BiSCaO Water against influenza A (H1N1), an enveloped virus, and *Feline calicivirus,* a nonenveloped virus, were evaluated using the fifty-percent tissue culture infectious dose (TCID_50_) method [19,27], which was performed by JFRL (Table 2). *Feline calicivirus* treated with BiSCaO Water was reduced to below the detection limit within 1 min, and influenza A was reduced from 6.0 to 1.7 (TCID_50_/mL) at 1 min compared with the control, and then reduced to below the detection limit after 5 min.

### 2.3. Deodorization Activity of BiSCaO Water, Dispersion, and Colloidal Dispersion

**Figure 2:**
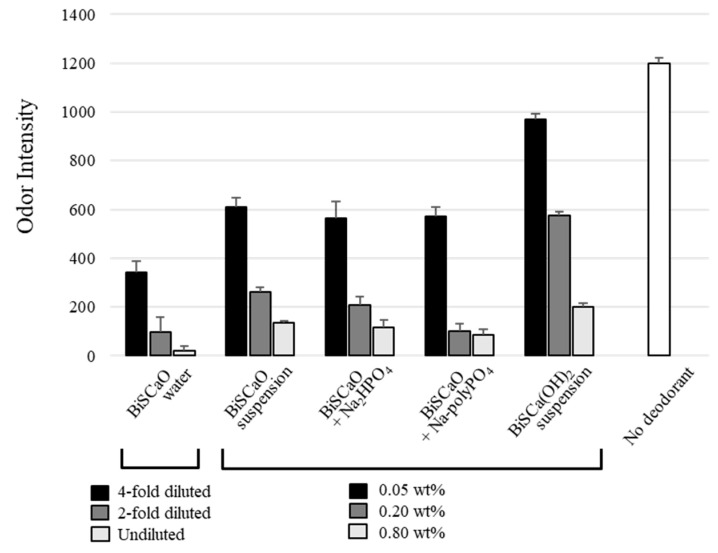
Efficacies of BiSCaO water, suspension, dispersion, and colloidal dispersions for deodorizing contaminated minced pork. Each deodorant was added to tainted pork meat on Petri dishes, then sealed in plastic bags for 1 h. The odor intensity was measured using a handheld odor meter. The experiments were repeated four times.

We evaluated and compared the deodorization efficacy of BiSCaO Water, BiSCaO and BiSCa(OH)_2_ suspensions, and BiSCaO dispersion and colloidal dispersion using tainted pork meat as a malodorous material. Tainted pork meat was mixed with each deodorant, then placed on Petri dishes and sealed in plastic bags for 1 h. The odor intensity was then measured using a handheld odor meter. All deodorants tested exhibited concentration-dependent deodorization effects. BiSCaO Water had the highest deodorization efficacy at each concentration tested; BiSCa(OH)_2_ suspensions were less efficient. BiSCaO suspension, dispersion, and colloidal dispersion exhibited intermediate efficacies (Figure 2).

### 2.4. Microbicidal Efficacy of BiSCaO Water, Suspension, Dispersion, and Colloidal Dispersion

**Figure 3:**
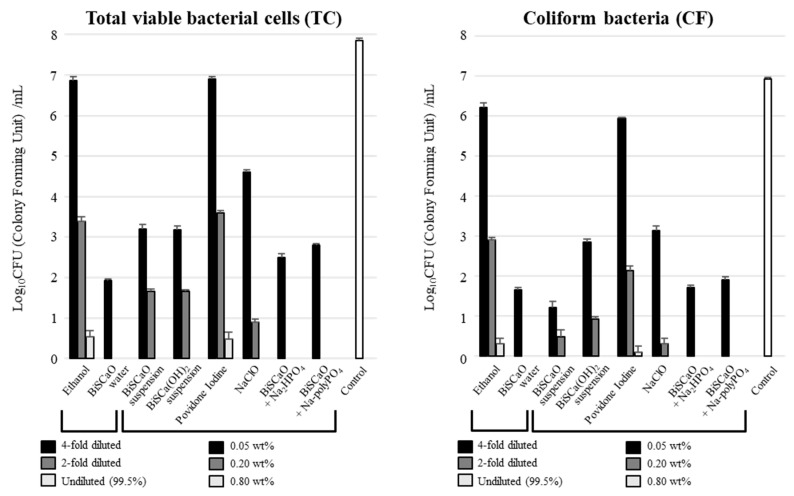
Bactericidal activity of disinfectants against a contaminated suspension comprising normal bacterial flora. Total viable bacterial cells (TC) (left) and coliform bacteria (CF) (right) released from the contaminated suspension in each sample were measured as CFU/mL. BiSCaO Water, dispersion, and colloidal dispersion had high microbicidal activities. Plating and counting were performed as a set of 4 technical replicates (n = 4).

We investigated the microbicidal efficacy of BiSCaO Water, suspension, dispersion, and colloidal dispersion against a contaminated suspension comprising normal bacterial flora, compared to other antiseptics/disinfectants. Equal volumes of each disinfectant and the contaminated suspension were mixed well and incubated at room temperature for 15 min, then the number of colony forming units (CFU/mL) per sample was determined. During incubation of bathtub water with 10% Dulbecco’s Modified Eagle’s Medium (DMEM) + bovine serum albumin (BSA) (0.1 wt%) at 37 °C for 24 h, the TC and CF values increased from 100 ± 45 CFU/mL and 65 ± 30 CFU/mL to 7.8 ± 1.5 (×10^7^) CFU/mL and 6.6 ± 1.8 (×10^6^) CFU/mL, respectively. The CFU/mL for TC and CF following treatment with undiluted (final 2-fold diluted) and 2-fold diluted (final 4-fold diluted) BiSCaO Water, and 0.8 and 0.2 wt% (final 0.4 wt% and 0.1 wt%) of BiSCaO dispersion and colloidal dispersion were below the detection limit, whereas low counts of TC and CF remained viable following treatment with 0.05 wt% of BiSCaO Water, dispersion, and colloidal dispersion (Figure 3). In contrast, some TC and CF remained viable following treatment with undiluted ethanol, 2-fold diluted, and 4-fold diluted (final 2-fold diluted, 4-fold diluted, and 8-fold diluted) ethanol, and 0.8, 0.2, and 0.05 wt% (final 0.4, 0.1, and 0.025 wt%) povidone iodine. The microbicidal activities of BiSCaO and BiSCa(OH)_2_ (suspension) and NaClO against TC and CF were intermediate between that of BiSCaO Water and povidone iodine, and no CFU were detectable following treatment with high concentrations of 0.8 wt% (final 0.4 wt%). Plating and counting were performed as a set of 4 technical replicates (n = 4).

**Figure 4:**
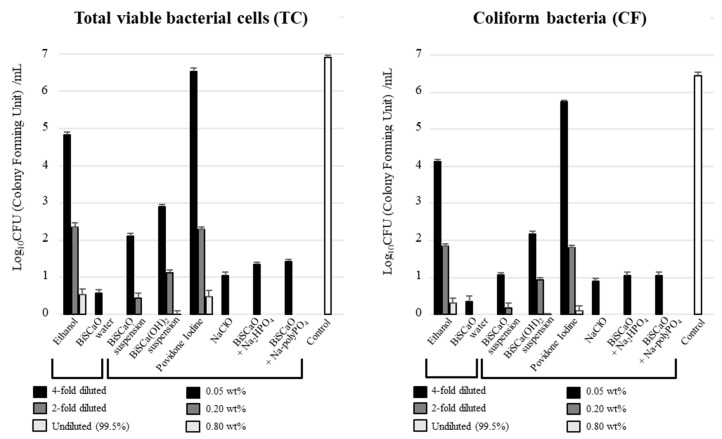
Disinfection efficacy using wood pieces. TC (left) and CF (right) released from the contaminated wood piece in each sample were measured as CFU/mL. BiSCaO Water, dispersion, and colloidal dispersion had high microbicidal activity. Plating and counting were performed as a set of 4 technical replicates (n = 4).

The number of colony forming units (CFU/mL) per sample was determined after disinfecting contaminated wood pieces with TC values of 8.8 ± 2.2 (×10^6^) and CF values of 5.4 ± 1.6 (×10^6^). The CFU/mL following treatment with undiluted and 2-fold diluted BiSCaO Water and 0.8 and 0.2 wt% of NaClO, BiSCaO dispersion, and colloidal dispersion were below the detection limit for both TC and CF, whereas low counts of TC and CF remained viable following treatment with 4-fold diluted BiSCaO Water and 0.05 wt% of NaClO, BiSCaO dispersion, and colloidal dispersion (Figure 4). Low counts of TC and CF remained viable following treatment with undiluted, 2-fold diluted, and 4-fold diluted ethanol; 0.8, 0.2, and 0.05 wt% BiSCa(OH)_2_; and povidone iodine. BiSCaO (suspension) had a microbicidal activity against TC and CF intermediate between that of BiSCaO Water and povidone iodine: the TC and CF counts were below the detection limit following treatment with a high concentration of 0.8 wt% BiSCaO (suspension).

### 2.5. Sterilization by BiSCaO Water Sprayed on a Mask Surface

**Figure 5:**
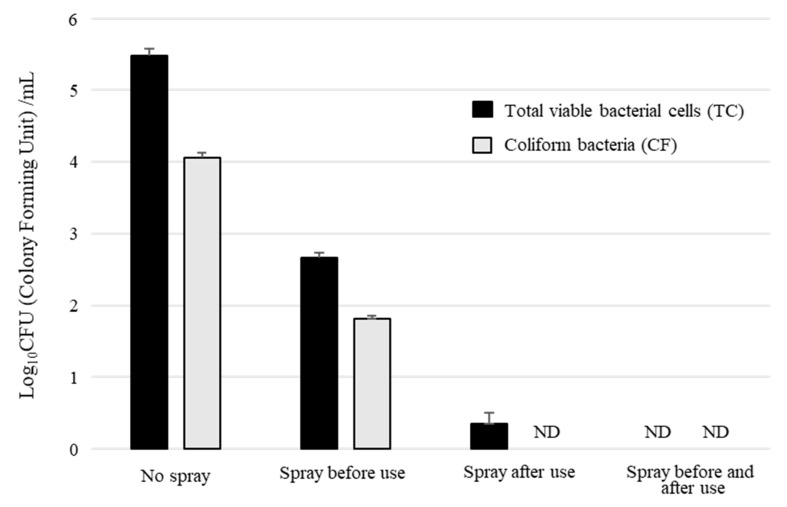
Sterilization of mask surfaces by spraying BiSCaO Water. Surgical masks coated with dry BiSCaO Water generated by spraying BiSCaO Water and drying had strong antimicrobial activity. BiSCaO Water sprayed on the contaminated surface of a surgical mask resulted in almost complete disinfection. The experiments were repeated four times.

A suspension (100 μL) containing 6.7 ± 1.2 (×10^6^) CFU/mL of TC and 5.4 ± 1.4 (×10^5^) CFU/mL of CF was inoculated on nonwoven surgical masks and dried or left wet, then BiSCaO Water was sprayed on some of the masks and air dried. The TC and CF of sprayed and unsprayed masks were about 72 (×10) and 90, and 48 (×10^4^) and 12 (×10^3^), respectively (Figure 5). Thus, dry white-powder-coated nonwoven surgical masks had strong antimicrobial activity. Furthermore, the spraying of BiSCaO Water on the contaminated surface of a surgical mask resulted in almost total disinfection. No change in quality was observed in the surgical mask after spraying BiSCaO ten times. This result suggested that surgical masks may be reused multiple times following disinfection with BiSCaO Water.

## 3. Discussion

Scallop shell is composed of CaCO_3_ and is converted to CaO when heated above 800 °C. According to the manufacturer, BiSCaO is prepared by heating shell powder at 1450 °C for 4 h to obtain over 99.6% CaO, grinding using a dry grinder, followed by cooling in a vacuum chamber and vacuum packing. The produced fine CaO powder, BiSCaO, has an average particle diameter of about 6 μm [25]. Both BiSCaO and BiSCa(OH)_2_ are poorly water-soluble under alkaline conditions. The generated precipitates in suspensions of high concentrations of BiSCaO and BiSCa(OH)_2_ can result in a significant loss of CaO and the plugging of spray nozzles. A methodology is therefore needed to prepare BiSCaO and BiSCa(OH)_2_ dispersions without precipitates. We previously reported that the addition of phosphate compounds such as phosphoric acid (H_3_PO_4_), trisodium phosphate (Na_3_PO_4_), Na_2_HPO_4_, or sodium dihydrogen phosphate (NaH_2_PO_4_) to BiSCaO or BiSCa(OH)_2_ suspensions results in the formation of dispersions [25]. Furthermore, BiSCaO and BiSCa(OH)_2_ colloidal dispersions can be prepared by mixing with Na-polyPO_4_ or Na-triPO_4_ as flocculent agents. Two layers quickly form a supernatant, and flocculants/precipitates composed of polymeric colloidal calcium phosphate [26]. The present study showed that BiSCaO Water (pH > 12.7), in addition to BiSCaO dispersion and colloidal dispersion, has microbicidal activity against various pathogenic bacteria, including *S. aureus* (a Gram-positive bacterium) (Table 1) and viruses such as influenza A (H1N1) as an enveloped virus and *Feline calicivirus* as a nonenveloped virus (Table 2).

BiSCaO Water, which is colorless and transparent with a pH > 12.7, was sprayed and dried on smooth metal or plastic surfaces and provided a white powder coating. Scanning electron microscopy (SEM) images of drying BiSCaO Water showed microparticles (1–2 μm) connected with each other (Figure 1a,b). Cryo-SEM observations indicated that CaO nanoparticles (100–200 nm) aggregated to generate larger BiSCaO particles (400–800 nm) in BiSCaO Water (Figure 1c,d).

We examined the deodorizing and bactericidal activities of BiSCaO Water, dispersion, and colloidal dispersion against TC and CF under various conditions such as particle size and concentration. BiSCaO Water, dispersion, and colloidal dispersion containing smaller particles and at higher concentration showed higher deodorization and microbicidal activities against TC and CF, probably due to higher Brunauer–Emmett–Teller (BET)-specific surface areas (data not shown). We anticipate that differences in the BET-specific surface areas of BiSCaO Water, dispersion, and colloidal dispersion influenced their deodorizing and microbicidal activities. Cryo-SEM showed that the nanoparticles in BiSCaO dispersion, colloidal dispersion, and BiSCaO Water were 160–200 nm [25], 160–300 nm [26], and 100–200 nm in diameter, respectively.

The hydration of CaO generates a strong base and is the primary mechanism for the deodorization and microbicidal activities of BiSCaO dispersion. The CaO content of BiSCaO is much higher than that of BiSCa(OH)_2_, and this suggested that BiSCaO Water, suspension, dispersion, and colloidal dispersion showed higher deodorizing and microbicidal activities than BiSCa(OH)_2_ because of the higher pH. However, our preliminary study showed that BiSCaO exhibited higher activity than NaOH solution at the same pH (data not shown). This suggests that alkalinity alone is not responsible for the deodorizing and microbicidal property of BiSCaO. Rather, we suggest that the microbicidal action is due to the reducing activity of BiSCaO, and that the high disinfection activity of BiSCaO is due to the OH− concentration of the thin water layer formed around BiSCaO particles being higher than in the bulk solvent [3,12]. Furthermore, active radical species generated from magnesium oxide and BiSCaO may also contribute to strong disinfection activity [3,12], as supported by a multiparameter flow cytometry study conducted by Hewitt et al. [29]. Although high pH is certainly the main contributor to the deodorizing and microbicidal activity of BiSCaO, active radical species generated from BiSCaO may be an alternative microbicidal factor.

The recent worldwide epidemic of coronavirus disease (COVID-19) due to a newly discovered coronavirus is causing a crisis [27,30]. The World Health Organization (WHO) recommends “to ensure that environmental cleaning and disinfection procedures are followed consistently and correctly. Thoroughly cleaning environmental surfaces with water and detergent and applying commonly used hospital-level disinfectants such as NaClO are effective and sufficient procedures.” [27]. Some antiseptics/disinfectants, such as ethanol and NaClO, show significant activity towards SARS-CoV-2 by breaking the envelope of virus. However, they are cytotoxic to cellular and organic components, and high concentrations are required for antiseptic/disinfection activity [31,32,33]. Furthermore, chlorine-derived compounds are ineffective in the presence of organic materials [16,30]. Therefore, antiseptics/disinfectants that can decrease the bacterial bioburden without harmful side effects and environmental disruption are essential for environmental hygiene and public health. The character of BiSCaO Water for having virucidal activity against an enveloped-type virus (Table 2) may be valuable for the limitation of the spread of respiratory viruses such as COVID-19, although it is necessary to study additional microbicidal activity including coronavirus SARS-CoV-2.

Recommendations for personal protective equipment (PPE), including face masks such as surgical masks or N95 respirators, are necessary for the protection of health-care personnel [34]. Recommendations on face masks vary across countries, and the use of masks increases substantially once local epidemics begin, including the use of masks in community settings. However, this increased use of face masks by the general public exacerbates the global supply shortage of face masks, sending prices soaring [35]. In this study, spraying BiSCaO Water on the contaminated surface of a surgical mask resulted in almost complete elimination of the test microbes without changing the quality of the mask. There is little possibility that dry microparticle-aggregates blow up and/or suck in, since nanoscale CaO particles in BiSCaO Water aggregate to generate larger BiSCaO particles and the microparticles were composed of CaCO_3_ produced by interaction of Ca^2+^ and CO_2_. This result suggested that face masks may be reused multiple times by sterilization with BiSCaO Water, which could contribute to their safe and economical reuse, rather than disposal after a single use. However, our preliminary experiments show that the potency of BiSCaO Water does not have a long-term effect on the spraying surface, therefore the spray may need to be performed both at the start of use and after use (Figure 5).

## 4. Materials and Methods

### 4.1. Preparation and Characterization of BiSCaO Water

According to the manufacturer, scallop shell powders were heated at 1450 °C for 4 h, then ground using a dry super grinder (Nano Jetmizer NJ-300-D; Aishin Nano Technologies Co. Ltd., Saitama, Japan), followed by cooling in a vacuum chamber. This provided BiSCaO dry powder with particle diameters of 3–9 μm (average 6 μm). This was purchased from Plus Lab Corp., Kanagawa, Japan. According to the manufacturer, the content of CaO in this BiSCaO preparation is 99.6%. BiSCa(OH)_2_ was obtained from Scallow, Kohkin Inst. Co. Ltd., Tochigi, Japan and had a dry-powder-particle diameter of 10–100 μm (average 46 μm). The CaO and Ca(OH)_2_ contents were <5% and >90%, respectively.

According to the manufacturer, BiSCaO Water was prepared by adding 100 g of BiSCaO to 1100 mL chilled clean water (<10 °C), gently mixing, and standing for 30 min. The supernatant (1000 mL) was collected and transferred to a 100-L water tank. Another chilled 1000 mL of clean water was gently poured and mixed onto the remaining BiSCaO precipitate and the supernatant was collected in the tank. This process was repeated fifty times. The produced BiSCaO water (total 100 L) was colorless and transparent with a pH of about 12.75, and the BiSCaO water is now commercially available from Plus Lab Corp.

Scanning electron microscope (SEM) images of dry powder were obtained by osmium metal coating using a neo-osmium coater (Neoc-STB; Meiwafosis Co., Ltd., Tokyo). The surface structure of each dry powder was observed using a field-resolved scanning electron microscope (JSM-6340F; JEOL Ltd. Tokyo, Japan). For cryo-SEM, samples were frozen in liquid nitrogen, then knife-cut and observed using a JEOL JSM7100F SEM (JEOL Ltd., Tokyo, Japan) under vacuum conditions at −90 °C. The accelerating voltage was 10 KV, and the detection signal was a backscattered electron image.

### 4.2. Assay of the Disinfection Activity of BiSCaO Water against Pathogenic Microbes In Vitro

We evaluated the bactericidal activities of BiSCaO Water towards *E. coli* (NBRC 3972), *E. coli* O-157:H7 (ATCC 43895), *P. aeruginosa* (NBRC 13275), *Salmonella* (NBRC 3313), and *S. aureus* (NBRC 12732). Each bacterial suspension was prepared in soybean-casein digest broth with lecithin and polysorbate (SCDLP) liquid and agar medium. The assays were performed by the Japan Food Research Laboratory (JFRL; Tokyo, Japan). Briefly, 0.1 mL of each bacterial suspension (107–108 cells/mL) was added to 10 mL of BiSCaO Water. The mixture was stirred and then left at room temperature for 1, 5, and 15 min to allow the bacteria to interact with BiSCaO. At each time point, the mixture was ten-fold diluted with SCDLP medium to terminate the interaction. The mixtures were subjected to ten-fold serial dilution with phosphate-buffered saline (PBS) onto SCDLP agar plates (8.5 cm). The number of CFU was determined after incubating at 35 °C for 24 h.

To evaluate the virucidal activities of BiSCaO Water, *Feline calicivirus* (F-9; ATCC VR-782) and human influenza A virus (H1N1; ATCC VR-1465) were used and assayed using SCDLP medium and the TCID_50_ method. The assays were performed by JFRL. Briefly, viral suspension in PBS (100 μL) was added to 1-mL BiSCaO Water. The mixture was stirred and then left at room temperature for 1, 5, and 15 min to allow the virus to interact with BiSCaO. At each time point, the mixture was ten-fold diluted with 10% fetal bovine serum containing DMEM to terminate the interaction. The mixtures were subjected to two-fold serial dilution with PBS in a 96-well cell culture plate sown with Crandell feline kidney (CRFK) cells for *Feline calicivirus* and Madin-Darby canine kidney (MDCK) cells for influenza A virus. The antiviral activity of BiSCaO Water was estimated as the TCID_50_ ratio of the BiSCaO-treated sample to the control (treated with clean water).

### 4.3. Deodorization Activity of BiSCaO Water

The addition of 0.05, 0.2, and 0.8 g of BiSCaO and BiSCa(OH)_2_ to 100 mL of clean water, followed by rotary mixing, generated 0.05, 0.2, and 0.8 wt% BiSCaO and BiSCa(OH)_2_ suspensions, respectively. Next, 0.04, 0.12, and 0.6 wt% of Na_2_HPO_4_ (FUJIFILM Wako Pure Chemical Corp., Osaka, Japan) for BiSCaO dispersion and Na-polyPO4 (FUJIFILM Wako Pure Chemical Corp.) for BiSCaO colloidal dispersion were added to 0.05, 0.2, and 0.8 wt% BisCaO water suspension, respectively, then rotary mixed to prepare each BiSCaO dispersion and colloidal dispersion. Ethanol (99.5%; FUJIFILM Wako Pure Chemical Corp.) was used and diluted with clean water.

Five grams of tainted pork meat was mixed with 10 mL of each deodorant, then placed on Petri dishes and sealed in plastic bags (7 × 10 cm) for 1 h. The odor intensity was measured using a handheld odor meter (OMX-SRM; Shinyei Technology Co. Ltd., Hyogo, Japan).

### 4.4. Microbicidal Efficacy of BiSCaO Water

The microbicidal efficacy of BiSCaO water against suspensions and wood pieces contaminated with high normal bacterial flora was evaluated and compared with the actions of ethanol, BiSCaO and BiSCa(OH)_2_ suspensions, povidone iodine, NaClO, BiSCaO dispersion, and colloidal dispersion. Various concentrations of povidone iodine and NaClO were prepared by the dilution of 7-wt% Isodine (Meiji Seika Pharma Co., Ltd., Tokyo, Japan) and 1-wt% NaClO (Yoshida Pharmaceutical Corp., Tokyo, Japan) with clean water. The concentrations of NaClO were confirmed as residual chlorine levels using ClO (HClO and ClO^-^) -selective test papers (high concentration, 25–500 ppm; low concentration, 1–25 ppm; Kyoritu Check Laboratory Corp., Tokyo, Japan).

Suspensions contaminated with normal bacterial flora were prepared by incubating bathtub water with 10% DMEM + BSA (0.1 wt%) at 37 °C for 24 h [11,12]. Ten milliliters of each disinfectant were added to 10 mL of the contaminated suspension, mixed well, and incubated at room temperature for 15 min. To prepare contaminated wood pieces, five wood pieces (1.2 cm × 1.2 cm × 0.2 cm) were added to 40 mL of the contaminated suspension and incubated at 37 °C for 24 h, then rinsed with clean water [12,24]. For the disinfection assay of contaminated wood pieces, a wood piece was added to 10 mL of each disinfectant, vortexed gently for 15 min, and then TC and CF were released from the contaminated wood piece in 10 mL of clean water by vigorous vortexing for 2 min.

To count the number of CFU, aliquots (1 mL of each mixture) were gently poured into individual Petri dishes containing prealiquoted portions of simple and easy dry medium for TC or CF (Nissui Pharmaceutical Co., Ltd., Tokyo, Japan) [11,12,25,26], and the plates were incubated for 24 h in a 37 °C incubator (A1201; IKUTA Sangyo Co., Ltd., Ueda, Nagano, Japan). Plating and counting were performed as a set of 4 technical replicates (n = 4).

### 4.5. Sterilization by BiSCaO Water Sprayed on Mask Surfaces

Suspension contaminated with 6.7 ± 1.2 (×10^6^) CFU/mL of TC and 5.4 ± 1.4 (×10^5^) CFU/mL of CF (100 μL total) was inoculated and dried for 30 min at room temperature on a round area (2-cm diameter) of the surface of a surgical mask (surgical mask ST; Utsunomiya Seisaku Co. Ltd. Osaka, Japan). “Spray before use” was conducted by spraying about 0.5 mL of BiSCaO Water on the round area and drying for 3 h at room temperature. “Spray after use” was conducted by spraying about 0.5 mL of BiSCaO Water on the inoculated round area and drying for 3 h at room temperature. The inoculated area of each mask was resected, and then TC and CF were released from the contaminated sections into 10 mL of clean water by vigorous vortexing for 2 min. To count the CFU, aliquots (1 mL of each mixture) were gently poured into individual Petri dishes containing prealiquoted portions of simple and easy dry medium for TC or CF, and the plates were incubated for 24 h in a 37 °C incubator. Plating and counting were performed as a set of 4 technical replicates (n = 4).

## 5. Conclusions

BiSCaO Water contains nanoparticles (100–200 nm) and is colorless and transparent, with a pH > 12.7. BiSCaO Water completely eliminated various pathogenic bacteria within 15 min and viruses within 5 min in in vitro assays. Furthermore, BiSCaO Water exhibited higher deodorization of tainted pork meat and higher microbicidal efficacy using suspensions contaminated with normal bacteria compared to ethanol, BiSCaO and BiSCa(OH)_2_ suspensions, povidone iodine, NaClO, BiSCaO dispersion and colloidal dispersion. The results showed that BiSCaO Water has excellent deodorization and disinfection activities for pathogenic microbes, potentially for COVID-19 measures.

## Figures and Tables

**Table 1:** Assay of the activity of BiSCaO Water against pathogenic microbes in vitro.

Bacterial Strain	0 min	1 min	5 min	15 min
*E. coli*	(CFU)	(CFU)	(CFU)	(CFU)
	―	3.6 × 10^5^	<10 *	<10 *
*E. coli* (control)	6.7 × 10^5^	―	―	6.1 × 10^5^
*E. coli* (O157:H7)	―	6.4 × 10^5^	4.6 × 10^2^	<10 *
*E. coli* (O157:H7) (control)	6.9 × 10^5^	―	―	6.6 × 10^5^
*P. aeruginosa*	―	1.4 × 10^4^	<10 *	<10 *
*P. aeruginosa* (control)	2.7 × 10^5^	―	―	4.0 × 10^5^
*Salmonella*	―	2.1 × 10^4^	<10 *	<10 *
*Salmonella* (control)	7.1 × 10^5^	―	―	7.7 × 10^5^
*S. aureus*	―	1.1 × 10^5^	1.1 × 10^4^	<10 *
*S. aureus* (control)	3.4 × 10^5^	―	―	4.7 × 10^5^

<10 *: nondetected, ―: not determined. CFU: colony forming units.

**Table 2:** Assay of the activity of BiSCaO Water against an enveloped virus and a nonenveloped virus.

Virus Strain	0 min	1 min	5 min	15 min
*Feline calicivirus*		<1.5 *	<1.5 *	<1.5 *
	(TCID_50_/mL)	(TCID_50_/mL)	(TCID_50_/mL)	(TCID_50_/mL)
*Feline calicivirus* (control)	6.7	―	―	7.0
*Influenza A* (H1N1)		1.7	<1.5 *	<1.5 *
*Influenza A* (H1N1) (control)	6.0	―	―	6.0

<1.5 *: nondetected, ―: not determined. TCID_50_: tissue culture infectious dose.
